# Comprehensive view on chemotherapy-free management of acute myeloid leukemia by using venetoclax in combination with targeted and/or immune therapies

**DOI:** 10.1038/s41420-025-02678-4

**Published:** 2025-08-13

**Authors:** David Kegyes, Andrei Tat, Alin Stefan Vizitiu, Daiana Vazar-Tripon, Radu Ilie, Adrian Bogdan Tigu, Diana Cenariu, Anamaria Bancos, Sabina Iluta, Ciprian Jitaru, Madalina Nistor, Radu Tomai, Diana Gulei, Mihnea Zdrenghea, Hermann Einsele, Gabriel Ghiaur, Carlo M. Croce, Ciprian Tomuleasa

**Affiliations:** 1 Department of Personalized Medicine and Rare Diseases, Medfuture Institute for Biomedical Research – Department of Hematology, Iuliu Hatieganu University of Medicine and Pharmacy, Cluj-Napoca, Romania; 2 Department of Oncology, Bistrita Emergency Hospital, Bistrita, Romania; 3 Department of Hematology, Ion Chiricuta Cancer Center, Cluj Napoca, Romania; 4 Department of Internal Medicine II, Julius Maximilians University, Wurzburg, Germany; 5 Department of Leukemia, Sidney Kimmel Comprehensive Cancer Center, The Johns Hopkins University, Baltimore, USA; 6 Department of Cancer Biology and Genetics, The Ohio State University, Columbus, OH USA

**Keywords:** Adaptive clinical trial, Drug development

## Abstract

A hallmark of cancer biology is resistance to apoptosis. BCL-2 is an anti-apoptotic molecule that is being overexpressed in several myeloid diseases, such as acute myeloid leukemia and myelodysplastic syndromes, but also in several lymphoid cancers, such as acute lymphoblastic leukemia, chronic lymphocytic leukemia, non-Hodgkin lymphomas and multiple myeloma. Venetoclax (VEN) is a BCL-2 small molecule inhibitor. Data about its structure, biochemical characteristics and in vitro efficacy against several blood cancer cell lines were first reported in 2013. Shortly after, the first clinical trials reported that single-agent VEN provides no long-term survival benefits. In contrast, when used in combination, VEN led to significantly improved outcomes and eventually to its first US FDA approvals in 2018. As the modern approach to treating hematological malignancies are the chemotherapy-free regimen, in the current manuscript, we provide a comprehensive view on all available therapies that are considered to be chemotherapy-free, with a special emphasis on acute myeloid leukemia (AML), where phase I-III clinical trials have provided the most data.

## Facts

- Venetoclax brought a major breakthrough in the clinical management of AML
- Chemotherapy-free regimens are associated with better patient reported outcomes
- Venetoclax alone does not always provide long-term survival benefits, but in association with immune therapies and/or targeted therapies, the outcomes are far better than current standard-of-care

## Open questions

- Will chemotherapy-free regiments completely replace current chemotherapy-based regimens?
- Will venetoclax alone or in combination with immune therapies and/or targeted therapies represent the backbone of future chemotherapy-free alternatives?

## Introduction

A hallmark of cancer biology is resistance to apoptosis [1]. BCL-2 is an antiapoptotic molecule that is being overexpressed in several myeloid diseases such as acute myeloid leukemia (AML) and myelodysplastic syndromes (MDS), but also in several lymphoid cancers such as acute lymphoblastic leukemia (ALL), chronic lymphocytic leukemia (CLL), non-Hodgkin lymphomas, and multiple myeloma (MM) [2–6]. AML is a blood cancer characterized by the clonal proliferation of immature myeloid precursors, leading to impaired hematopoiesis in the bone marrow [7]. Evaluation of the performance status of the patient and a thorough morphologic, immunophenotypic, and molecular analysis are essential to classify and risk-stratify AML patients in order to design individualized treatment plans [8]. While the “7 + 3” cytarabine-anthracycline-based intensive chemotherapy (IC) regimen has long been considered the gold standard, advances in genomic profiling have transformed AML treatment with the introduction of multiple targeted therapies. FLT3-inhibitors like midostaurin or gilteritinib and IDH1- or IDH2-inhibitors, such as ivosidenib and enasidenib are already FDA-approved agents integrated in the daily practice. Additionally, gemtuzumab ozogamicin (GO) is a feasible option for patients with CD33-positive AML, especially in favorable-risk cases and also as an effective cytoreductive agent [9, 10]. Venetoclax (VEN) is a BCL-2 small-molecule inhibitor. Data about its structure, biochemical characteristics and in vitro efficacy against several blood cancer cell lines have been first published in 2013 [6]. Shortly after, the first clinical trials reported that single-agent VEN provided no long-term survival benefits [11]. In contrast, when used in combination, VEN led to significantly improved outcomes and trials leading to its US FDA approval, starting from 2018, have been summarized in Table 1. Tables 2–4 outline relevant clinical trials of VEN-based, chemo-free combinations which hold the potential to transform the therapeutic landscape for AML in the future. All chemotherapy-containing regimens were excluded from our analysis. Our review provides a thorough and visionary overview of current clinical status, unknowns and drawbacks of VEN therapy in AML. We summarize immunotherapy and targeted therapy combinations to provide state-of-the-art clinical insights for clinicians and highlight important gaps in current knowledge regarding several treatment aspects, such as which combination is best for specific patient subgroups, the optimal number of cycles, minimal residual disease (MRD)-tailored decisions, and the optimal timing for treatment discontinuation.

**Table 1:** Currently FDA-approved combinations for hematologic malignancies.

Disease	Drug Combination	Disease stage	Year of US FDA Approval	Trials that led to approval
Acute myeloid leukemia (AML)	VEN + azacitidine	newly-diagnosed (age 75 years or older)	2018 (accelerated) 2020 (regular)	phase 2 M14-358 (NCT02203773) phase 3 VIALE-A (NCT02993523)
	VEN + decitabine	newly-diagnosed (age 75 years or older)	2018 (accelerated) 2020 (regular)	phase 2 M14-358 (NCT02203773)
	VEN + LDAC	newly-diagnosed (age 75 years or older)	2018 (accelerated) 2020 (regular)	phase 2 M14-387 (NCT02287233) phase 3 VIALE-C (NCT03069352)
Chronic Lymphocytic Leukemia (CLL)	VEN + rituximab	R/R who received at least one prior therapy	2018	phase 3 MURANO (NCT02005471)
	VEN + obinutuzumab	newly-diagnosed	2019	phase 3 CLL14 (NCT02242942)
Myelodysplastic Syndrome (MDS)	VEN + azacitidine	newly-diagnosed intermediate-, high-, and very high–risk	2021 (breakthrough designation only)	phase 2 M15-531 study (NCT02942290)

**Table 2:** HMA+VEN combinations for acute myeloid leukemia.

Drug class combined with VEN	Drug combination	NCT ID	Phase	*N*	Status	Patient Population	Regimen
Hypomethylating agents	AZA + VEN	NCT02203773 M14-358 [12, 13]	I	145	Completed	N/D	- determination of optimal VEN doses (400 versus 600 versus 800 mg - 400 mg led to lowest incidence of adverse events with similar efficacy compared to higher doses
		NCT02993523 (VIALE-A) [15]	III	431	Completed	N/D	- AZA + VEN *versus* AZA + placebo - both elderly (≥75 years) and young (> 18 years) - unfit for intensive chemotherapy - no limited number of 28D cycles - SC or IV AZA 75 mg/m^2^, once daily from D1 to D7 - 100 mg oral VEN on D1 of cycle 1 followed by venetoclax 200 mg, once orally on D2 of cycle 1 and 400 mg on D3 to D28 - assessment of Health-Related QoL - assessment of percentage of transfusion independence defined as >56 D
		NCT05554406 (MyeloMATCH) [31]	II	335	Recruiting	N/D	- AZA + VEN versus liposomal 7 + 3 versus liposomal 7 + 3 + VEN or liposomal cytarabine + standard daunorubicin - fit, intensive chemotherapy-eligible patients - adverse risk AML - Two cycles of 28D AZA + VEN induction - MRD-negativity as a primary outcome measure
		NCT03573024 [23]	II	36	Recruiting	N/D	- young, fit, intensive chemotherapy-eligible patients - up to 4 cycles of 28D - SC or IV AZA 75 mg/m^2^, once daily from - the first 4 cycles 400 mg VEN then higher, 600 mg VEN if tolerated in maintenance, in case of MRD-positivity
		NCT05904106 (VINCENT) [24]	II	146	Recruiting	N/D	- AZA + VEN *versus* 7 + 3 + gemtuzumab ozogamicin - NPM1-mutated AML - fit, intensive, chemotherapy-eligible patients (age 18–70 years) - SC or IV AZA once daily on D1–D7 - oral, once daily VEN on D1–D28
		NCT04867928 (GIMEMA AML2521) [57]	II	35	Recruiting	N/D	- MRD-positive NPM1-mut AML - bridging therapy to transplant - 400 mg oral VEN on D1 to D28 - SC or IV AZA 75 mg/m^2^, once daily from D1 to D7 - 28D cycles - Six cycles then proceed to alloHSCT
		NCT05833438 (VENAZA-5S)	II	45	Recruiting	N/D	- assessment of AZA + VEN in older patients unfit for intensive chemotherapy - 5D of AZA instead of 7D per cycle to reduce toxicities - up to 6 cycles
		NCT03466294 [44]	II	42	Not Recruiting	N/D	- AZA + VEN induction until MRD-negative followed by VEN maintenance - MRD threshold not mentioned
		NCT03013998 (Beat AML) [64]	II	166	Recruiting	N/D	- direct comparison of 14 days versus 28 days VEN induction - unfit for intensive chemotherapy
		NCT03941964 [47]	III	60	Completed	N/D	- AZA/DEC + VEN in outpatient settings - SC or IV AZA once daily on D1–D7 - oral, once daily VEN on D1–D28
		NCT04905810	II	20	Recruiting	R/R	- AZA + VEN induction in patients with prior exposure to a hypomethylating agent given for other hematologic disorders, such as myelodysplastic syndromes - 28-day cycles until disease progression or unacceptable toxicities - SC or IV AZA once daily on D1–D7 - oral, once daily VEN on D1–D28
		NCT04062266 (QUAZAR AML-001) [41]	II	50	Recruiting	N/D	- reduced dose AZA + VEN maintenance for MRD-positive patients following either intensive chemotherapy or AZA + VEN induction - reduced dose (50 mg/m2) SC AZA on D1–D5 - oral VEN once daily on D1–D14 - up to 24 cycles
		NCT05554419 (ERASE)	II	184	Not Recruiting	N/D	- direct comparison of cytarabine alone *versus* cytarabine + VEN versus cytarabine + daunorubicin versus AZA + VEN to convert MRD-positivity before alloHSCT - two cycles
		NCT04128501	II	125	Recruiting	N/D	adverse risk AML AZA + VEN post-HSCT maintenance up to 12 cycles SC once daily AZA on D1–D5 oral VEN once daily on D1–D7, every 28D
		NCT04809181	II	95	Recruiting	N/D	- AZA + VEN maintenance in MRD-positive patients after alloHSCT - up to 24 cycles - assessment of OS - assessment of the incidence of transplant-related complications following AZA + VEN
		NCT05404906	II/III	124	Recruiting	N/D	- comparison of single-agent AZA maintenance *versus* AZA + VEN in first CR in young adults following intensive chemotherapy induction and consolidation independent of MRD-status - SC AZA on D1–D5 - oral VEN once daily on D1–D14 - eight cycles of 28D
		NCT04161885 (VIALE-T)	III	465	Not Recruiting	N/D	- AZA + VEN post-HSCT maintenance - SC AZA once daily on D1–D5 for up to 6 cycles - oral, once daily VEN on D1–D28 for up to 24 cycles - MRD assessment with a threshold of 10^‑^^3^ by flow cytometry
	CC-486 (oral AZA) + VEN	NCT05287568 [48, 49]	I	22	Recruiting	R/R	- assessment of safety and maximum tolerated dose of oral AZA (CC-486) + VEN induction - oral AZA on D1–D14 - oral VEN on D1–D28
		NCT04887857 (OMNIVERSE) [48, 49]	I	6	Completed	N/D R/R	- assessment of safety of oral AZA (CC-486) + VEN induction - oral AZA on D1–D14 - oral VEN on D1–D28 or D1–D21
		NCT04102020 (VIALE-M)	III	112	Not Recruiting	N/D	- oral AZA + oral VEN maintenance therapy following intensive chemotherapy induction
	DEC + VEN	NCT02203773 M14-358 [12, 13]	I	145	Completed	N/D	- determination of optimal VEN doses (400 versus 600 versus 800 mg
		NCT03941964 [47]	III	60	Completed	N/D	- assessment of DEC + VEN in outpatient setting - IV DEC once daily on D1–D5 - oral, once daily VEN on D1–D28
		NCT04752527 [16]	II	42	Not Recruiting	N/D	- assessment of DEC + VEN in young (<59 years old) patients - adverse risk AML - IV DEC once daily on D1–D5 - oral, once daily VEN on D1–D28
		NCT05177731 [32]	III	188	Recruiting	N/D	- direct comparison of DEC + VEN induction versus 7 + 3 standard chemotherapy in young adults - 2 cycles - IV DEC once daily on D1–D5 - oral, once daily VEN on D1–D28
		NCT03404193 [22]	II	235	Not Recruiting	N/D R/R	- 10-day IV DEC on D1–D10 instead of the classical 5-day regimen - older patients, unfit for intensive chemotherapy - up to 24 cycles of 28 days - oral, once daily VEN on D1–D28 of cycle 1 and D1–D21 for subsequent cycles
		NCT06285136 [129]	II	40	Not Recruiting	R/R	- reduced duration, 3-day DEC administration - fit patients aged 18–65 years - IV 20 mg/m^2^ DEC once daily on D4–D6 - oral 400 mg VEN once daily on D1–D14 with a 3-day dose ramp up
		NCT06073730 [40]	III	154	Not Recruiting	N/D	- reduced duration, 3-day DEC administration - unfit for intensive chemotherapy - IV 20 mg/m^2^ DEC once daily on D4–D6 - oral 400 mg VEN once daily on D1–D14 with a 3-day dose ramp up
		NCT04476199 (VEN-DEC GITMO) [56]	II	100	Completed	N/D	- elderly but transplant-eligible high- and intermediate-risk AML patients - two cycles of 28 days - IV 20 mg/m^2^ DEC once daily on D1–D5 - oral 400 mg VEN once daily on D1–D28 with a 3-day dose ramp up
		NCT06046313	II	120	Recruiting	N/D	- older patients, unfit for intensive chemotherapy - up to 24 cycles of 28D - ultra-low dose DEC (6 mg/m^2^) once daily on D1–D10 - oral, once daily VEN on D1–D21
		NCT06129734	I/II	20	Not Recruiting	N/D R/R	- DEC + VEN post-HSCT maintenance - once weekly SC low-dose DEC 5 mg/m^2^ - once weekly oral 400 mg VEN - up to 1 year
		NCT05184842 [42]	II	85	Recruiting	N/D	N/D AML patients ineligible to IC once weekly SC low-dose DEC 0.2 mg/kg once weekly oral 400 mg VEN
		NCT04763928	II	101	Not Recruiting	N/D	- secondary AML to myeloproliferative neoplasms - IV 20 mg/m^2^ DEC once daily on D1–D5 - oral 400 mg VEN once daily on D1–D28 with a 3-day dose ramp up - the 28D cycles continue until disease progression or intolerable toxicities
		NCT04905810	II	20	Recruiting	R/R	- DEC + VEN induction in patients with prior exposure to a hypomethylating agent given other other hematologic disorders, such as myelodysplastic syndrome - 28-day cycles until disease progression or unacceptable toxicities - IV DEC once daily on D1–D5 - oral, once daily VEN on D1–D28
	ASTX727 (oral DEC) + VEN	NCT04657081 [50]	I/II	188	Completed	N/D	- unfit for intensive chemotherapy - use of cedazuridine to improve oral DEC bioavailability - safety assessment
		NCT04746235 [51]	II‘	100	Recruiting	N/D R/R	- unfit for intensive chemotherapy - use of cedazuridine to improve oral DEC bioavailability - up to 24 cycles - oral DEC once daily on D1–D3 - oral VEN once daily on D1–D21
		NCT03306264 (ASCERTAIN) [53]	III	227	Completed	N/D	- direct comparison of ASTX727 versus IV DEC - unfit for intensive chemotherapy - use of cedazuridine to improve oral DEC bioavailability
		NCT04975919 [54]	II	20	Not Recruiting	R/R	- 10-day ASTX727 + VEN induction followed by 5 day/cycle maintenance - up to 24 cycles - use of cedazuridine to improve oral DEC bioavailability - extramedullary AML also eligible - oral DEC once daily on D1–D10 - oral VEN once daily on D1–D21 or D28
		NCT04817241	I/II	55	Not Recruiting	N/D	- direct comparison of ASTX727 + VEN versus “7 + 3” - younger (18–65 years), non-FLT3 mutated AML - use of cedazuridine to improve oral DEC bioavailability - up to 12 cycles - oral DEC once daily on D1–D4 versus D1–D5 - oral VEN once daily on D1–D21 versus D1–D28
		NCT05799079	II	51	Recruiting	R/R	- both young and elderly AML patients relapsed post-HSCT - use of cedazuridine to improve oral DEC bioavailability - oral DEC once daily on D1–D5 - oral 400 mg VEN once daily on D1–D28 with a 3-day dose ramp up - the 28D cycles continue until disease progression or intolerable toxicities
		NCT05010772 [55]	I	125	Recruiting	N/D	- ASTX727 + VEN maintenance in first complete remission before alloHSCT - patients are allowed to receive either intensive or lower intensity therapies as induction - use of cedazuridine to improve oral DEC bioavailability - up to 24 cycles - oral DEC once daily on D1–D3 - oral VEN once daily on D1–D5

*Allo-HSCT* allogeneic hematopoietic stem cell transplantation, *AZA* azacytidine, *VEN* venetoclax, *D* day, *QoL* quality of life, CR complete remission.

**Table 3:** Outcomes and toxicity of HMA + VEN depending on VEN treatment duration.

VEN treatment duration	ORR (%)	Complete response rate (%)	MRD-negativity (%)	mPFS (months)	mOS (months)	2-months mortality (%)	Grade 3 or higher Neutropenia (%)	Grade 3 or higher anemia or RBC transfusion rate (%)	Grade 3 or higher thrombocytopenia or platelet transfusion rate (%)
7 days									
Willekens et al. [38]	79	72	70	6.5	11.2	6	48	84	62
14 days									
Aiba et al. [130]	75	50	50	not reached	not reached	0	87.5	12	12.5
Karrar et al. [33]	-	68	65	-	18.6	-	45	-	48
Kanaya et al. [131]	-	67.7	-	-	not reached	-	67.4	-	-
Ginosyan et al.[37]	-	27	-	-	6.1	-	-	-	-
Cui et al. [36]	69.4	66.7	-	12	17	30-day mortality 0%	36	44	25
21-days									
Karrar et al. [33]	-	66	72	-	21.3	-	39	-	38
Kanaya et al. [131]	-	51	-	-	14.1	-	78.4	-	-
Ginosyan et al. [37]	-	72	-	-	17.6	-	-	-	-
Mirgh et al. [132]	-	75	-	-	-	-	75	76.4	75
28 days									
VIALE-AStudy [12, 15]	64.7	38.8	42	9.9	14.7	30-day mortality 7%	43	28	46
Aiba et al. [130]	80	40	20	5.85	8.5	20	20	40	80
Karrar et al. [33]	-	62	81	-	13.2	-	42	-	41
Kanaya et al.[131]	-	38.9	-	-	10.8	-	50	-	-
Ginosyan et al. [37]	-	68	-	-	15.7	-	-	-	-
Mirgh et al. [132]	-	100	-	-	-	-	87.5	62.5	50

*ORR* overall response rate, *MRD* minimal residual disease, *mPFS* median progression-free survival, *mOS* median overall survival, *RBC* red blood cells.

**Table 4:** Venetoclax and targeted therapy combinations for acute myeloid leukemia.

Drug class combined with VEN	Drug combination	NCT ID	Phase	*N*	Status	Patient population	Regimen
FLT3- inhibitors	gilteritinib + VEN	NCT03625505 [60, 61]	I	61	Completed	R/R	- FLT3mut AML - 120 mg oral gilteritinib on D1–D28 - 400 mg oral VEN on D1–D28 - pharmacokinetic analysis
	gilteritinib + AZA + VEN	NCT05520567 [64]	I/II	70	Recruiting	N/D	- FLT3mut AML (ITD or D835 - elderly patients (>75 years old) unfit for intensive chemotherapy - assessment of the safety of the triplet - oral 80 mg gilteritinib on D1–D28 - IV or SC 75 mg/m^2^ AZA on D1–D7 - oral 400 mg VEN on D1–D28 with a 3-day dose ramp up
	gilteritinib + AZA + VEN	NCT04140487 [62]	I/II	55	Not Recruiting	N/D R/R	- FLT3mut AML (ITD or D835) - patients 18 years or older unfit for intensive chemotherapy - up to 24 cycles - oral AZA once daily on D1–D7 - oral 400 mg VEN once daily on D1–D28 with a 3-day dose ramp up - oral 80 mg gilteritinib once daily on D1–D28
	gilteritinib + AZA + VEN	NCT06317649 (Myelomatch MM1OA-EA02) [66]	II	147	Recruiting	N/D	- comparison of 80 mg oral gilteritinib once daily on D1–D28 *versus* D8-D21 - FLT3mut AML (ITD or D835) - elderly patients (>75 years old) unfit for intensive chemotherapy - two cycles of induction and maintenance up to 2 years with the same regimen - IV or SC AZA once daily on D1–D7 - oral 400 mg VEN once daily on D1–D28 with a 3-day dose ramp up
	gilteritinib + DEC + VEN	NCT03013998 (Beat AML S8) [64]	I/II	17	Recruiting	N/D	- patients aged >60 years, unfit for intensive chemotherapy - oral 80 mg gilteritinib on D1–D28 seemed the best, but multiple dose-levels have been tested - IV 20 mg/m^2^ DEC on D8–D1 - oral VEN 400 mg on D1–D28
	gilteritinib + ASTX727 (oral DEC) + VEN	NCT05010122	I/II	42	Recruiting	N/D R/R	- FLT3mut AML (ITD or D835) - 24 cycles + gilteritinib maintenance - oral ASTX727 once daily on D1–D5 - oral VEN once daily on D1–D21 - oral gilteritinib once daily on D1–D28
	quizartinib + AZA + VEN (VEN-A-QUI)	NCT04687761	I/II	84	Recruiting	N/D	- unfit for intensive chemotherapy - regardless of FLT3 mutational status - SC or IV AZA 75 mg/m^2^, once daily on D1–D5 and D8-9 (5 “on”, 2 “off”, 2 “on) - oral VEN once daily on D1–D28 - oral quizartinib once daily on D1–D5
	quizartinib + DEC + VEN	NCT03661307 [68]	I/II	73	Recruiting	N/D R/R	- FLT3-ITD mutant AML - two cycles - IV DEC once daily on D1–D10 - oral VEN once daily on D1–D14 - oral quizartinib once daily on D1–D28
	tuspetinib +VEN + - AZA	NCT03850574 (APTIVATE) [70, 71]	I/II	218	Recruiting	R/R	- safety assessment
IDH1/2 -inhibitors	ivosidenib + AZA + VEN	NCT03471260 [73]	I/II	96	Recruiting	N/D R/R	- IDH1m AML (R132) - unfit for intensive chemotherapy - SC AZA once daily on D1–D7 - oral VEN once daily on D1–D14 - oral ivosidenib once daily on D15–D28
	enasidenib + VEN	NCT04092179 [75]	I/II	27	Completed	R/R	- IDH2m AML (R140 or R172) - oral VEN once daily on D1–D28 - oral enasidenib once daily on D15–D28 - cycles continue until disease progression or intolerable toxicities
	ivosidenib or enasidenib + ASTX727 (oral DEC) + VEN	NCT04774393 [76]	I/II	84	Recruiting	R/R	- unfit for intensive chemotherapy - bilineage leukemia or isolated extramedullary AML also eligible - oral ASTX727 once daily on D1–D5 - oral VEN once daily on D1–D14 - oral ivosidenib or enasidenib on D1–D28 - cycles continue until disease progression or intolerable toxicities
	enasidenib + ASTX727 + VEN (MyeloMatch subtrial)	NCT06672146	II	93	Not yet recruiting	N/D	- unfit for intensive chemotherapy - oral enasidenib once daily on D1–D28 - oral ASTX727 once daily on D1–D5 - oral VEN once daily on D1–D28
	olutasidenib + DEC + VEN	NCT06445959	I/II		Recruiting	N/D R/R	- unfit for intensive chemotherapy - safety and pharmacokinetic assessment
	crelosidenib (LY3410738, dual IDH1/IDH2 inhibitor) + AZA + VEN	NCT04603001	I	260	Not Recruiting	N/D R/R	- IDH1 R132, IDH2 R140 or IDH2 R172 mutated AML - 75 years or older, unfit for intensive chemotherapy - safety and pharmacokinetic assessment
	enasidenib + AZA *versus* AZA + VEN	NCT05401097 (I-DATA) [72]	II	125	Recruiting	N/D	- sequential IDH-inhibitor + AZA followed by AZA + VEN *versus* AZA + VEN followed by IDH-inhibitor + AZA - CR has to be reached in 3 AZA + VEN cycles and 5 IDH-inhibitor + AZA cycles - SC AZA once daily on D1–D5 for up to 6 cycles - oral, once daily VEN on D1–D28 for up to 24 cycles
Menin -inhibitors	revumenib + VEN	NCT06284486 [83]	II	8	Recruiting	N/D	- efficacy of revumenib + VEN in clearance of MRD following high-intensity or low-intensity therapies - NPM1mt, or KMT2Ar, or NUP98r AML in CR with MRD ≥ 0.1% - oral VEN once daily on D1–D14 - oral revumenib once daily on D1–D18 - cycles continue until disease progression or intolerable toxicities
	revumenib + AZA + VEN	NCT06177067	I	24	Recruiting	R/R	- KMT2A, NUP98, NPM1-mutated AML - age ≤30 years
	revumenib + ASTX7272 (oral DEC) + VEN	NCT05360160 (SAVE) [80]	I/II	43	Recruiting	N/D R/R	- KMT2Ar, or NUP98r, or NPM1-mutated AML - unfit for intensive chemotherapy - oral revumenib twice daily on D1–D28 - oral ASTX727 once daily on D1–D5 - oral VEN once daily on D1–D14
	ziftomenib + AZA + VEN	NCT05735184 (KOMET-007) [78]	I	212	Recruiting	N/D R/R	- comparison of 7 + 3 + ziftomenib versus ziftomenib + AZA + VEN in N/D patients - KMT2A, NPM1-mutated AML - young patients also included - oral revumenib on D8-D28 in cycle 1, D1–D28 in the other cycles - IV or SC AZA on D1–D5 - oral VEN on D1–D28
		NCT06397027 (ZiVa) [79]	I	22	Not yet recruiting	R/R	- pediatric and young adult patients (2–21 years) - oral revumenib on D8-D28 in cycle 1, D1–D28 in the other cycles - IV or SC AZA on D1–D5 - oral VEN on D1–D14
	bleximenib (JNJ-75276617) + AZA + VEN	NCT05453903	I	150	Recruiting	R/R	- KMT2A, NPM1-mutated AML - secondary AML also accepted - safety assessment
	emilumenib (DS-1594b) + AZA + VEN	NCT04752163	I/II	17	Completed	R/R	- in arm A patients enrolled irrespective of mutational status - safety assessment
p53- reactivators and MDM2 antagonists	idasanutlin + VEN	NCT02670044 [85]	II	88	Completed	R/R	- unfit for intensive chemotherapy - oral idasanutlin on D1–D5 - oral VEN on D1–D28
		NCT04029688	I/II	38	Completed	R/R	- young adults (age <30 years) - oral idasanutlin on D1–D5 - oral VEN on D1–D28
	navtemadlin + DEC + VEN	NCT03041688	I	58	Recruiting	R/R	- wild-type TP53, adverse risk AML - 4 cycles of induction then maintenance - oral navtemadlin on D1–D7 in both induction and maintenance - IV DEC on D1–D10 in induction and D1–D5 in maintenance - oral VEN on D1–D21 in induction and D1–D14 in maintenance
	siremadlin (HDM201) + VEN	NCT03940352 [86]	I	52	Not Recruiting	R/R	- wild-type TP53, adverse risk AML - unfit for intensive chemotherapy - safety and pharmacokinetics assessment - oral siremadlin on D1–D5 - IV or SC AZA on D1–D7 - oral VEN on D1–D28
	siremadlin (HDM201) + AZA + VEN	NCT05155709 [87]	I	14	Not Recruiting	N/D	- wild-type TP53, adverse risk AML - unfit for intensive chemotherapy - safety and pharmacokinetics assessment - oral siremadlin on D1–D5 - IV or SC AZA on D1–D7 - oral VEN on D1–D28
	eprenetapropt + AZA + VEN	NCT04214860 [88]	I	51	Completed	R/R	- TP53-mutated AML - oral eprenetapropt on D1–D4 - IV or SC AZA on D1–D7 - oral VEN on D1–D28
Cyclin -dependent kinase (CDK) -inhibitors	alvocidib + VEN	NCT03441555 [90]	I	36	Completed	R/R	- IV alvocidib on D1–D3 - oral VEN on D1–D28 - safety and pharmacokinetics assessment
	fadraciclib + VEN	NCT04017546	I	14	Completed	R/R	- safety and pharmacokinetics assessment
	voruciclib + VEN	NCT03547115 [91]	I	100	Recruiting	R/R	- safety and pharmacokinetics assessment
	QHRD107 + AZA + VEN	NCT06532058 [92]	I	53	Recruiting	R/R	- safety and pharmacokinetics assessment
	RVU120 + AZA + VEN	NCT06191263 (RIVER-81)	II	98	Recruiting	R/R	- 21-day cycles - oral RV120 on D1–D13 - SC or IV AZA on D1–D7 - oral VEN on D1–D14 - safety and pharmacokinetics assessment
	SLS009 (GFH009) + AZA + VEN	NCT04588922	I/II	135	Recruiting	R/R	- adverse risk, ASXL1, BCOR, EZH2, SF3B1, SRSF2, STAG2, U2AF1 and ZRSR2 mutated AML - safety and pharmacokinetics assessment - IV SLS009 twice a week - SC or IV AZA on D1–D7 - oral VEN on D1–D28
	HC-7366 + AZA + VEN	NCT06285890	I	18	Not Recruiting	R/R	- GCN2 (general control nonderepressible 2) kinase inhibitor - safety and pharmacokinetics assessment - oral HC-7366 on D1–D28 - SC or IV AZA on D1–D7 - oral VEN on D1–D28
Cerebron ligase inhibitors	selinexor + VEN	NCT03955783	I	78	Completed	R/R	- adverse risk AML - oral selinexor on D1, D8, D15, D22 - oral VEN on D1–D28
	selinexor + AZA + VEN	NCT05736965 [94]	II	58	Recruiting	N/D	- unfit for intensive chemotherapy - oral selinexor on D3, D10, D17 - SC or IV AZA on D1–D3, D8–D9, D15–D16 - oral VEN on D3–D14
	selinexor + AZA + VEN	NCT05736978	II	58	Recruiting	N/D	- unfit for intensive chemotherapy - patients will get selinexor based on MRD-results on D14 - oral selinexor on D15 and D22
	eltanexor + VEN	NCT06399640	I	60	Not Recruiting	R/R	- eltanexor 5 days a week for 14, 21, or 28 days - oral VEN on D1–D14
	regorafenib + AZA + VEN	NCT06454409	I/II	20	Not Recruiting	R/R	- multi-kinase inhibitor - unfit for intensive chemotherapy - 12 cycles - oral regorafenib on D1–D21 - IV AZA on D1–D7 - oral VEN on D1–D21
	trametinib + AZA + VEN	NCT04487106 [96]	II	21	Completed	N/D R/R	-MEK-inhibitor - unfit for intensive chemotherapy - 24 cycles - oral trametinib on D1–D28 - SC or IV AZA on D1–D7 - oral VEN on D1–D21
	cobimetinib + VEN	NCT02670044 [97]	II	88	Completed	R/R	- MEK-inhibitor - unfit for intensive chemotherapy - oral cobimetinib on D1–D28 - oral VEN on D1–D21
	mivebresib + VEN	NCT02391480 [98]	I	128	Completed	R/R	- BET-inhibitor
	emavusertib + VEN	NCT04278768 [101]	I/II	336	Recruiting	R/R	- IRAK4-inhibitor - FLT3m, SF3B1 or U2AF1 mutated AML - oral emavusertib on D1–D21 - oral VEN on D1–D21 - 28-day cycles
	cirtuvivint + ASTX727 (oral DEC) + VEN	NCT06484062	I	48	Not Recruiting	R/R	- CDC-like kinase (CLK) inhibitor.
	CCS1477 (inobrodib) + AZA + VEN	NCT04068597	I/II	250	Recruiting	R/R	- EP300/CBP bromodomain inhibitor
	danvatirsen + VEN	NCT05986240	I	24	Recruiting	R/R	- STAT-inhibitor
	OPB-111077 + DEC + VEN	NCT03063944 [104]	I	37	Completed	N/D R/R	- STAT-inhibitor - unfit for intensive chemotherapy - OPB-111077 on D1–D28 - IV DEC on D4–D8 - oral VEN on D4–D28

*AZA* azacytidine, *VEN* venetoclax, *D* day, *QoL* quality of life, *CR* complete remission.

## Hypomethylating agents (HMA)

**Fig. 1:**
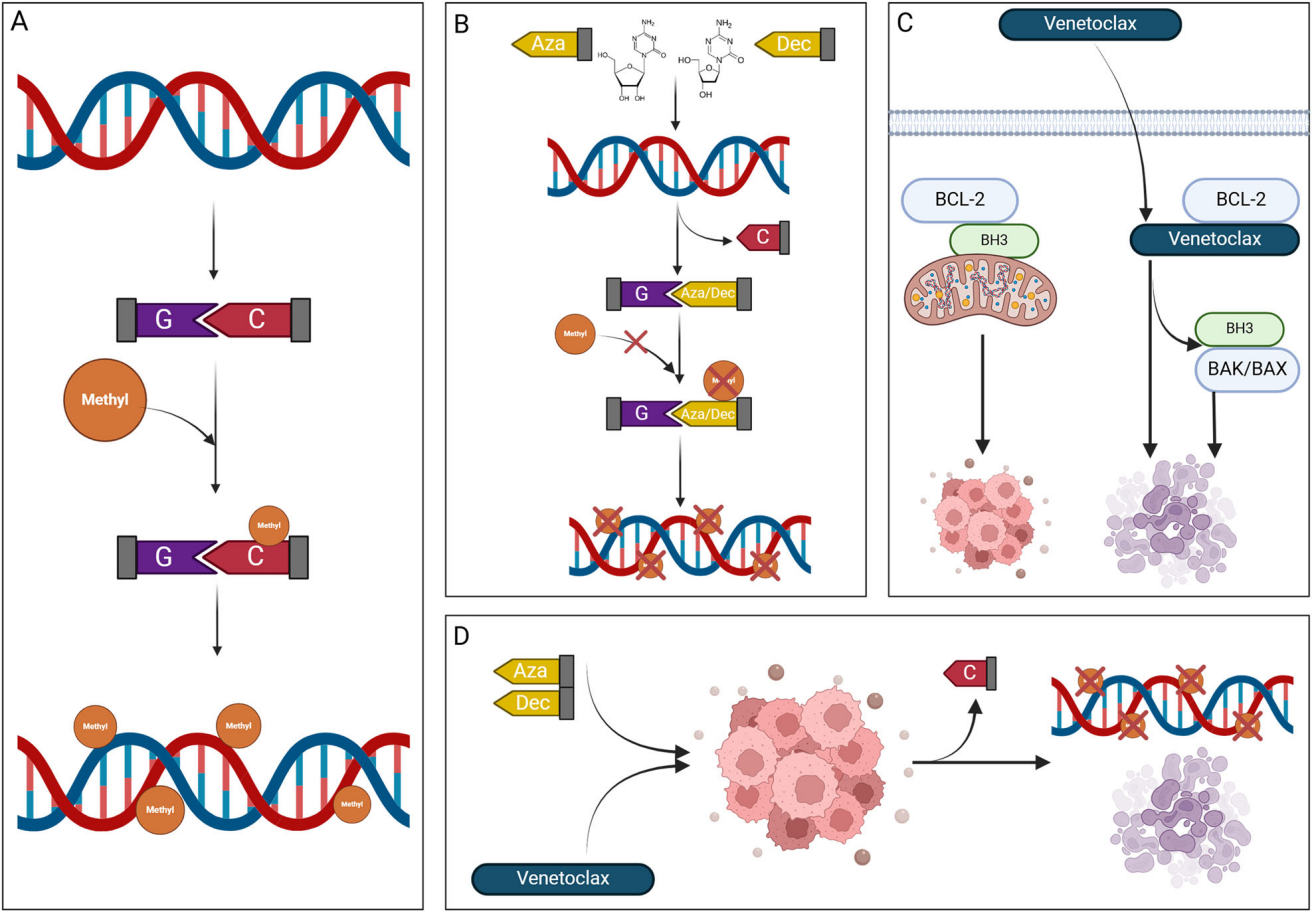
The synergistic effects of HMA and VEN. **A** The methylation as an epigenetic process in the DNA, leading to methyl cytosine (5-mC), is used as regulators of different promoters and protein-encoding genes. **B** Cytosine analogs— azacytidine (Aza) and decitabine (Dec)—mimic cytosine but have an extra nitrogen instead of a carbon in the 5’ position. During cell division, the analogs act as demethylating agents, replacing wild-type cytosine, thus the methyl group cannot be incorporated into DNA, leading to global demethylation. **C** The overview of the mechanism of action of venetoclax, binding to BCL-2 and letting BH3-peptides free to bind to BAK/BAX, triggering apoptosis. **D** The expected outcome of a combination therapy using venetoclax and HMA, with hypomethylated tumor cells and high apoptosis rates.

The combination of HMA, such as azacitidine (AZA) and decitabine (DEC), with VEN has been shown to work synergistically, their mechanism of action being described in Fig. 1.

The first question that may arise when combining HMA with VEN is whether efficacy is maintained across all age groups. The phase 2 M14-358 (NCT02203773) and phase 3 VIALE-A (NCT02993523) studies were the first to demonstrate that 7 days of AZA or 5 days of DEC combined with 28 days of VEN significantly outperform AZA + placebo in older adults (≥75 years) with newly-diagnosed (N/D) AML, particularly those unfit for intensive chemotherapy [12, 13]. A recent pooled analysis of VIALE-A demonstrated consistent safety and efficacy data across all elderly cohorts (75–79 years, 80–84 years, ≥85 years) [14]. A recent update also indicated a survival advantage in younger (<75 years) patients with IDH1-, IDH2-, or NPM1-mutant AML. This trend, however, has not been observed in the case of FLT3- or TP53 mutations [15]. Younger, adverse-risk AML patients, including those with complex karyotypes or TP53-mutations achieved, however, higher rates of response with 5 days of DEC + VEN compared to AZA + VEN based on the results of the phase 2 NCT04752527 [16]. Thus, genetic subtypes, rather than age, are more powerful predictors of response and outcomes. IDH1-mutated AML treated with HMA + VEN compared favorable to IDH2-mutations, with the mention that the small sample size and the enrichment for NPM1-mutations in the IDH1 cohort may have been confounding factors [17]. ASXL1 mutations without the presence of adverse karyotype or TP53 mutations also predicted better responses in N/D patients [18]. In contrast, a novel survival prediction model indicated a 1-year survival <1% in patients with ASXL1, RAS or TP53 mutations that failed frontline HMA + VEN vs 42% in the absence of these [19]. N/KRAS, KMT2A, and SF3B1 mutations were also associated with worse outcomes [20].

A second important clinical question is whether longer treatment duration might improve the depth and duration of response. Beyond genetic risk factors, monocytic differentiation identified by morphology or flow cytometry has also been associated with resistance to HMA + VEN (CR rates of 26.7% in monocytic-like vs 80% in non-monocytic-like subtypes, *P* < 0.001) [21]. A novel approach of 10-day DEC instead of the classical 5-day regimen, however, improved response rates of monocytic leukemia, reaching an overall response rate (ORR) of 86% with 68% of these being sustained at 1 year. Also, substantial improvements in response rates have been observed with 10-day DEC + VEN for RUNX1, FLT3, and TP53 mutations with an impressive ORR for the overall population of 89% and median OS of 18.1 months (95% CI, 10—not reached) [22]. Longer, 10-day administration of DEC was well tolerated in older subjects, without any therapy-related mortality and a 1-year progression-free survival (PFS) of 84% [17].

Randomized, controlled, trials comparing IC with HMA + VEN in N/D AML are lacking. The phase 2 NCT03573024 concluded that IC and AZA + VEN in non-favorable risk, young (<60 years) AML patients led to comparable ORR (50 vs 61%). However, a lower median number of days admitted to the hospital (30 vs 7.5, *P* < 0.0001), lower median units of platelet (11 vs 4, *P* = 0.0076) or red blood cell transfusions (9 vs 3.5, *P* = 0.0068) and a lower incidence of infections (93 vs 39%, *P* = 0.0071) favored AZA + VEN [23]. The complex morphologic, genetic, and molecular subtypes of AML make the selection of an ideal treatment and the comparison of IC with lower intensity regimens extremely difficult. The VINCENT phase 2 trial is currently enrolling N/D, NPM1-mutated, FLT3-wild-type subjects to assess AZA + VEN vs 7 + 3 + GO [24]. A retrospective study that examined younger (60–75 years) AML patients treated with HMA + VEN did not show any difference in CR rates and median overall survival (OS), in case of NPM1, TP53, and FLT3-ITD mutant AML compared to IC [25]. Similar outcomes have been reported in N/D AML patients (age ≥60 years) carrying an IDH1- or IDH2-mutation [26]. Higher ORR with AZA + VEN compared to IC, however, have reported with mutations in RUNX1 (82% vs 45%, *P* = 0.003), chromatin-cohesin genes (ASXL1, EZH2, BCOR, STAG2) (77 vs 47%, *P* = 0.005) or myelodysplasia-related genes (86 vs 43%, *P* = 0.001), with higher overall MRD-negative CR rates in the AZA + VEN arm (46 vs 19%, *P* < 0.001) [27]. A potential benefit of IC over HMA + VEN was linked to favorable-risk AML, normal cytogenetics and RAS pathway mutations [25]. Patients aged 60–75 years with very-high risk cytogenetics, such as inv(3)(q21.3q26.2) t(3;3)(q21.3;q26.2)/GATA2:MECOM(EVI1), t(3q26.2;v)/MECOM(EVI1)-rearranged, complex karyotype or monosomal karyotype, but without a TP53 mutation, achieved longer median OS when treated with IC (14 months, 95% CI 10-30), compared to AZA + VEN (8.0 months, 95% CI 5.2-15) [28]. Monosomy 5 or deletions in chromosomes 5 or 7 have also been linked to a longer median OS when treated with IC vs AZA + VEN. However, when adjusted by age to the 60–75 age range, there was no statistically significant difference in survival [29]. In case of previously untreated secondary AML, AZA + VEN seems to be superior to the “7 + 3” regimen, but there was no difference in efficacy compared to CPX-351 [30]. The myeloMATCH phase 2 trial (NCT05554406), among others, is currently recruiting young (<60 years), adverse risk N/D AML patients to directly compare two cycles of induction with AZA + VEN vs liposomal “7 + 3” vs liposomal “7 + 3” + VEN vs liposomal cytarabine + standard daunorubicin [31]. Also, the efficacy of HMA + VEN in secondary AML patients with prior exposure to hypomethylating agents is currently under investigation in the phase 2 NCT04905810 trial. In case of DEC + VEN, the first interim results of the NCT05177731 phase 2 trial comparing it to standard “7 + 3” in young adults (<60 years) with N/D AML have also been published. DEC + VEN showed significantly higher complete response (CR) and MRD-negativity rates with a significantly decreased incidence of grade 3 adverse events in the intermediate- and adverse-risk AML group, especially for patients aged 40 and older (*P* < 0.001) [32].

Apart from the selection of an optimal regimen, the length of VEN-based induction therapies is an ongoing topic of discussion. Table 5 summarizes the results of several studies comparing the length of VEN therapy in N/D AML patients ineligible for IC. Karrar et al. reported that patients with one or more favorable-risk mutations (NPM1, IDH2, and DDX41) and without any adverse-risk features (TP53, FLT-ITD, and RUNX1) showed no significant difference in CR rates with 14 days vs 21 days vs 28 days of VEN exposure (92, 90, and 100%; *P* = 0.19). Similarly, in patients with unfavorable cytogenetics, CR rates were not substantially improved in those who received VEN for 28 days (44%) compared to 21 days (60%) or 14 days (22%) (*P* = 0.18). CR rates of TP53-mutated patients were also comparable (*P* = 0.81). Moreover, duration of response was similar in patients receiving VEN for 14 days (8.7 months, 95% CI 1–37) vs 28 days (8.3 months, 95% CI 1–5, *P* = 0.12) [33]. These shorter cycles might especially favor the outcomes of the older individuals.

**Table 5:** Venetoclax and immunotherapy combinations for acute myeloid leukemia.

Drug class combined with VEN	Drug combination	NCT ID	Phase	*N*	Status	Patient Population	Regimen
Checkpoint -inhibitors	pembrolizumab (anti-PD1) + AZA + VEN [105]	NCT04284787 [105]	II	76	Completed	N/D	- SC AZA once daily on D1–D7 - oral VEN once daily on D1–D28 - IV pembrolizumab on D8 of cycle 1, every 3 weeks on cycle 1–6 - induction/consolidation of total six cycles - maintenance up to 24 cycles
	pembrolizumab (anti-PD1) + DEC + VEN	NCT03969446	I	54	Recruiting	R/R	- unfit for intensive chemotherapy - 8 cycles of 42 days - IV pembrolizumab on D1 and D22 of every cycle - IV DEC on D1–D10 - oral VEN on D1–D14
	nivolumab (anti-PD1) + DEC + VEN	NCT04277442	I	13	Not Recruiting	N/D	-TP53m AML - 3 cycles followed by maintenance without DEC - IV nivolumab on D1 and D15 (except cycle when with only D15) - SC or IV DEC on D1–D10 - oral VEN on D1–D21
	avelumab (anti-PD-L1) + AZA + VEN	NCT03390296 [106]	I/II	50	Completed	R/R	- IV avelumab on D1 and D14 - SC or IV AZA on D1–D7 or D1–D5 + D8–D9 - oral VEN on D1–D21
	magrolimumab (anti-CD47) + AZA + VEN	NCT05079230 (ENHANCE-3)	III	378	Completed	N/D	- unfit for intensive chemotherapy - IV magrolimumab 1 mg/kg on D1 and D4, 15 mg/kg on D8, 30 mg/kg on D11, D15 then weekly for 5 weeks and every 2 weeks thereafter - SC or IV AZA on D1–D7 - oral VEN on D1–D28
		NCT04435691	II	110	Not Recruiting	N/D R/R	- unfit for intensive chemotherapy - 12 cycles - IV magrolimumab on D1, D4, D8, D11, D15, and D22 of cycle 1, D 1, D8, D15, and 22 of cycle 2, and D1 and D15 of cycle 3 and subsequent cycles - SC or IV AZA on D1–D7 - oral VEN on D1–D28
	SL-172154 (anti-CD47) + AZA + VEN	NCT05275439	I	160	Recruiting	R/R	- adverse risk AML - safety assessment
	AK117 (anti-CD47) + AZA + VEN	NCT06387420	I/II	180	Not Recruiting	R/R	- adverse risk AML - safety assessment
	6MW3211 (anti-CD47/anti-PD-L1) + AZA + VEN	NCT05448599	I/II	120	Recruiting	R/R	- fit AML patients (< 75 years) - 6MW322 once every second week - SC AZA on D1–D7 - oral VEN on D1–D28
	sabalolimab - MBG452 (anti-TIM3) + AZA + VEN	NCT04150029 (STIMULUS- AML1)	II	90	Not Recruiting	N/D	- unfit for intensive chemotherapy - 12 cycles
	LYT-200 (anti-galectin-9) + AZA/DEC + VEN	NCT05829226	I	90	Recruiting	R/R	- both fit and unfit patients eligible - IV LYT-200 once a week
Monoclonal antibodies	gemtuzumab ozogamicin (anti-CD33) + VEN	NCT04070768	I	18	Not Recruiting	R/R	- safety assessment
	gemtuzumab ozogamicin (anti-CD33) + AZA + VEN	NCT03390296 [106]	I/II	21	Completed	R/R	- gemtuzumab ozogamicin on D8 - SC or IV AZA on D1–D7 or D1–D5 + D8–D9 - oral VEN on D1–D21
	cusatuzumab (anti-CD70) + AZA + VEN	NCT04150887 (ELEVATE) [115]	I	61	Not Recruiting	N/D	- unfit for intensive chemotherapy - IV cusatuzumab on D3 and D17
		NCT06384261 (OV-AML-1231)	II	120	Not Recruiting	N/D	- unfit for intensive chemotherapy - IV cusatuzumab on D3 and D17 - direct comparison to AZA + VEN alone
	aplitabart (anti-DR5) + VEN	NCT04553692	I	430	Recruiting	N/D R/R	- death receptor 5-targeted therapy
	NP137 + AZA + VEN	NCT06150040	I/II	35	Recruiting	R/R	- nectin-1 directed therapy
Antibody- drug conjugate	tagraxofusp (SL-401) (anti-CD123) + AZA + VEN	NCT03113643 [118]	I	72	Recruiting	N/D R/R	- adverse risk AML - unfit for intensive chemotherapy - CD123-directed therapy - IV tagraxofusp on D1–D3 or D1–D5 - IV AZA on D1–D7 - oral VEN on D1–D21
	tagraxofusp (SL-401) (anti-CD123) + AZA + VEN	NCT06456463	II	72	Recruiting	N/D	- unfit for intensive chemotherapy - CD123-directed therapy - IV tagraxofusp on D4–D6 - IV AZA on D1–D7 - oral VEN on D1–D28
	pivekumab sunirine (IMGN632) + AZA + VEN	NCT04086264 [119]	I/II	218	Not Recruiting	N/D R/R	- CD123-directed therapy - both fit and unfit patients eligible - 21-day cycles - IMGN632 administered on D7 of each cycle
	lintuzumab- Ac225 + VEN	NCT03867682 [117]	I/II	38	Recruiting	R/R	- CD33-directed therapy - lintuzumab-Ac225 on D5 for 4 cycles - oral VEN on D1–D21 for 12 cycles - 28-day cycles with expansion to 42 days in case of cytopenias
Natural Killer (NK)-cell Engagers	SAR443579 + AZA + VEN	NCT06508489	I/II	18	Recruiting	N/D	- unfit for intensive chemotherapy - IV SAR443579 weekly - IV AZA on D1–D7 - oral VEN on D1–D28
Adoptive Cellular Therapies	cytokine-induced memory-like NK-cells + VEN	NCT06152809	I	10	Not Recruiting	N/D	- patients with adverse risk AML or mutations associated with VEN resistance - MRD+ patients in CR are eligible - safety assessment
	allogeneic NK-cells + AZA + VEN	NCT05834244 (ADVENT-AML)	I	32	Recruiting	R/R	- patients with adverse risk AML - unfit for intensive chemotherapy and HSCT

*AZA* azacytidine, *VEN* venetoclax, *D* day, *QoL* quality of life, *CR* complete remission.

A retrospective study with only 14 days of VEN showed improved PFS, longer OS and less toxicity than administration for ≥15-day [34]. Another retrospective analysis showed that patients with ≤14-day VEN exposure had significantly improved median PFS (15.8 months vs 8.7 months, *P* = 0.01) and OS (24.7 months vs 11.3 months, *P* = 0.006) compared to the ≥15-day arm. Also, a reduced median duration of VEN/cycle was associated with a significant decrease in risk of death (HR 0.18, 95% CI 0.07–0.48, *P* = 0.0007). Moreover, extended cycle intervals (≥35 days), to allow count recovery, were not linked to an increased risk of death (HR 0.59, 95% CI 0.27–1.26; *P* = 0.17). A significant survival benefit of reduced treatment duration has been observed in TP53-mutated AML (median OS 14.7 months vs 8.7 months; *P* = 0.026) [35]. Cui et al. confirmed that 14-day administration is comparable to outcomes of VIALE-A, and it is the first study to report that the FAB-M5 AML patients achieved a superior outcome with 14-day VEN compared to other subtypes [36]. In contrast, Ginosyan et al. reported that 21 days of VEN led to longer OS (6.1 months *vs* 17.6 months, HR 3.08, 95% CI, 1.10–8.59, *P* = 0.032) and lower relapse rates (HR 6.56, 95%, CI 2.37–18.2, *P* < 0.001) compared to the 14-day cohort [37]. Since results are controversial, more prospective, randomized trials are required before drawing any conclusions. The Beat AML phase 2 trial (NCT03013998), for instance, is currently recruiting patients to directly compare 14 days vs 28 days of VEN.

The most recent retrospective study conducted by Willekens et al. also compared 7-days VEN vs SOC VEN regimens (14 days or more) and found no difference in ORR, CR rate and MRD-negativity rate. The patients, however, tolerated more cycles (median of 6 vs 3, *P* < 0.01) with a lower median time between cycles (28–30 days vs 36–39 days). In terms of toxicity, early 8-week mortality was significantly decreased (6 vs 16%, *P* = 0.03) with a reduction in the need for platelet transfusions (62 vs 72%, *P* = 0.02). A prospective, phase 3 trial (SEVENAZA) has been initiated in order to compare efficacy and outcomes of the “7 + 7” VEN + AZA regimen [38]. As a conclusion, multiple factors might play a role in improving outcomes with the reduction of VEN treatment duration, such as reduced mortality or improved tolerance leading to a lower discontinuation rate, a lower rate of dose reductions and thus an increased HMA exposure. Also, a reduction of continuous BCL-2 inhibition might decrease intracellular adaptive mechanisms that may lead to drug resistance [39].

Another clinical trend is to reduce not just the dose of VEN, but also the exposure to HMA. The phase 2 VENAZA-5S (NCT05833438) trial is currently recruiting subjects to assess the safety and efficacy of a reduced 5-day AZA schedule in elderly, frail patients. Another study, NCT06073730, assessed the efficacy of a 3-day DEC and 14-day VEN induction followed by 2-day decitabine consolidation cycles in elderly patients unfit for IC. Preliminary data on 47 subjects suggests that 3-day DEC induction leads to superior outcomes and significantly less myelotoxicity than previously reported with 5-day or 10-day administration [40]. The phase 2 NCT06285136 trial investigated the same regimen but in younger patients (<65 years). Results seem really promising with 18 out of 23 patients reaching MRD-negativity [41]. NCT05184842 investigated 28-day VEN with low-dose decitabine (0.2 mg/kg weekly *vs* 20 mg/m^2^/day for 5 days). In this trial, the CR rates (61 vs 68%) and median OS (16.1 months vs 14.7 months) were comparable to those observed in VIALE-A [42].

MRD assessment is crucial for the evaluation of treatment responses and the prediction of relapse. Monitoring MRD allows for early intervention, MRD-guided decision-making is still limited to clinical trial settings. We identified several studies that assessed the prognostic value of different MRD measurement tools, optimal MRD threshold cut-offs or MRD-guided treatment discontinuation of HMA + VEN combinations. Importantly, MRD conversion in earlier HMA + VEN treatment cycles did not improve outcomes. MRD-negativity with a threshold of 10^−3^, regardless of the timepoint of conversion, however, has been a favorable prognostic factor, achieving OS of 34.2 months (95% CI, 27.7–44.0) vs 18.7 months (95% CI, 12.9–23.5) [15, 43]. MRD negativity after HMA + VEN in NPM1-mutations, determined by digital droplet PCR, did not impact OS or PFS. MRD negativity by flow, however, was predictive of better survival [44]. A retrospective study confirmed the efficacy of AZA + VEN reinduction in case of post-IC MRD, 19/27 of treated patients achieving MRD-negativity. NPM1 or FLT3-ITD mutations and an early MRD relapse (less than 12 months after CR) were not associated with subsequent MRD response failure following AZA + VEN [45]. Patients not reaching CR after one cycle of IC, however, seem to benefit more by a second cycle of IC than switching to HMA + VEN [46]. In case of relapse in the first 6 months after achievement of CR, the survival benefit of IC was not significant anymore, median OS being comparable (9 months for IC vs 6 months for HMA + VEN, *p* = 0.4). Age, ELN risk stratification, and cytogenetics did not significantly impact survival either [46]. MRD-guided discontinuation of AZA has also been tested in the phase 2 NCT03466294, without any benefits. Moreover, after azacitidine was stopped, its reintroduction in case of relapse could not bring a new response, or a decrease in MRD levels [44]. Thus, HMA + VEN maintenance therapy cycles seem to be crucial for durable responses. It is currently unknown whether decreasing HMA doses or lowering/increasing the recommended number of cycles is better to optimize the efficacy of the incidence of adverse events. Recent data suggests that lower doses of subcutaneous (SC) AZA (50 mg/m^2^) maintain efficacy while decreasing long-term toxicities. Patients with NPM1-, IDH1-, or IDH2-mutations showed a 2-year PFS of 79% (95% CI, 60–100) with no relapses occurring in the first year of maintenance. Mutations in FLT3, RAS, TET2, or DNMT3A were not significantly associated with differences in PFS [41]. The number of cycles in the clinical trials summarized by us ranges from six to 24. Most of these have not yet published any results.

Outpatient induction or maintenance with IV/SC HMA + VEN in the NCT03941964 trial reported that adverse events were consistent with those seen in inpatient settings and there were no safety concerns [47]. To facilitate outpatient administration, improve patient quality of life, and improve safety, all-oral HMA + VEN combinations have also been designed. CC-486, an oral formulation of AZA given for 14 days/cycle demonstrated comparable response rates with conventional IV or SC AZA in R/R AML patients in the phase 1 OMNIVERSE (NCT04887857) and NCT05287568 trials [48, 49]. CC-486 + VEN vs *CC-486 +* placebo as maintenance therapy for patients 18 years and older with N/D AML in first CR after IC is also tested in the phase III randomized, double-blind VIALE-M (M19-708) study (NCT04102020). ASTX727, oral DEC, +VEN is another all-oral option, both for N/D and R/R AML patients. The phase 1 NCT04657081 was the first trial to prove the safety of ASTX727 + VEN in older patients unfit for IC [50]. NCT04746235 is a phase 2 trial that confirmed the efficacy of this combination in elderly patients, with a median OS of 11.5 months (95% CI 9.1–16.6) in the frontline and 7.2 months (95% CI 6.3–NA) in the salvage therapy cohort. There was no significant difference in OS in younger patients compared to those aged 80 years or more (P = 0.825). Also, the same study demonstrated that the European Leukemia Net (ELN) risk classification published in 2022 did not effectively stratify patients in terms of OS (*P* = 0.84) [51]. Genetic groups, however, successfully predicted outcomes, TP53, N/KRAS and FLT3-ITD mutations being associated with a shorter duration of response [52]. The phase 3 ASCERTAIN (NCT03306264) study directly compared the pharmacokinetics and efficacy of ASTX727 vs IV DEC and found no significant difference in plasma concentrations and efficacy [53]. Efficacy of all-oral 5-day ASTX727 + 21-day VEN induction is directly compared with the standard “7 + 3” regimen in younger, fit patients in the phase 2 NCT04817241 trial. NCT04975919 enrolled R/R AML patients and administered 10 days of ASTX727 + 21 or 28 days of VEN/cycle and achieved a median OS of 9.1 months and CR in four of seven VEN-naive and 1 of 11 VEN-exposed patients suggesting that patients without VEN-based initial induction regimens may benefit more from this combination [54]. NCT05010772 demonstrated that the addition of VEN, gilteritinib or ivosidenib to ASTX727 is a feasible all-oral maintenance therapy approach [55].

Even in the era of targeted therapies, transplantation in eligible patients remains an essential factor in improving long-term prognosis. Whether VEN-based regimens are optimal as bridging therapies to transplant remains a critical clinical question. The phase 2 VEN-DEC GITMO (NCT04476199) demonstrated high efficacy and safety of two cycles of 5-day DEC + VEN as bridging to allotransplant [56]. A longer, 10-day DEC + VEN combination also proved to be an active salvage and bridging therapy in molecularly predefined R/R AML subpopulations, particularly in the case of NPM1, IDH1, IDH2, and FLT3-mutations [22]. The phase 2 GIMEMA AML2521 (NCT04867928) study enrolled NPM1-mutated MRD-positive AML patients following two cycles of IC. AZA + VEN led to the achievement of a molecular response in 80% of these patients. Therefore, this approach might be a feasible bridging therapy for transplant in the future [57]. The phase 2 ERASE study (NCT05554419) is currently enrolling patients to directly compare the efficacy in terms of MRD-conversion before undergoing allotransplantation of cytarabine alone vs cytarabine + VEN vs cytarabine + daunorubicin vs AZA + VEN. AZA + VEN proved to be safe and active in post-transplant R/R disease as well, with 57% (30/52) of patients reaching CR and 18.3% (9/52) achieving MRD-negativity [58]. Post-allotransplant maintenance with all-oral HMA + VEN is also currently being tested in NCT05799079.

## FLT3-inhibitors

Current guidelines do not recommend the administration of frontline FLT3-inhibitors in IC-ineligible patients. We identified no trial assessing the efficacy of midostaurin, a first-generation FLT3-inhibitor, with chemo-free VEN-based regimens. However, case series report efficacy of 21-day/cycle midostaurin + VEN in t(8;21)(q22;q22.1)/AML1-ETO) core binding factor-AML associated with KIT mutations, R/R to frontline HMA + VEN regimens [59].

Although no survival benefit of the second-generation FLT3-inhibitor gilteritinib + AZA combination has been shown in the NCT02752035 trial, improved efficacy was reported in NCT03625505 with gilteritinib + VEN in the case of R/R AML [60, 61]. Later, the NCT04140487 trial added AZA to the gilteritinib + VEN backbone which demonstrated modest survival in R/R patients, but significantly increased efficacy in N/D patients unfit for IC, with 25 of 27, reaching MRD-negativity by a FLT3, polymerase chain reaction (PCR)-based assay. However, OS and median PFS have not yet been reached and significant toxicities have been reported [62]. A retrospective analysis of the triplet in N/D patients reported a median OS of 28.1 months, outcomes being superior in case of non-FLT3-ITD mutations (median OS of 39.1 months vs 24.5 months). NRAS, KRAS, PTPN11, CBL, NF1, and BRAF mutations have been associated with relapses. Outcomes have not been influenced by age groups (≥75 years vs <75 years, *P* = 0.65) [63]. The phase 2 NCT05520567 trial is currently enrolling elderly N/D patients to confirm the efficacy of the triplet therapy in patients 75 or older. Beat AML S8 (NCT03013998) assessed gilteritinib with DEC + VEN, and the preliminary data showed high efficacy, with tolerable adverse events and no treatment-related mortality [64]. Also, an all-oral triplet therapy with ASTX727 proved to be safe and showed an ORR of 53% in R/R patients [65]. NCT05010122 is currently recruiting N/D patients to assess this all-oral combination. Therefore, currently available data suggest that the addition of 80 mg gilteritinib daily to VEN alone or AZA/DEC + VEN to earlier treatment phases leads to superior outcomes in FLT3-mutated AML patients. Optimal treatment duration with gilteritinib remains to be determined. The MyeloMATCH MM1OA-EA02 substudy (NCT06317649) is directly comparing 28-day vs 14-day administration of gilteritinib [66].

Another active second-generation FLT3-inhibitor tested with AZA + VEN or DEC + VEN is quizartinib. Preliminary data from the phase 1/2 VEN-A-QUI trial indicated comparable efficacy to gilteritinib-containing triplets in N/D patients regardless of their FLT3 mutational status. However, early toxicity and a case of early death have been observed, warranting further evaluation of the safety of this combination [67]. While FLT3-ITD mutations led to worse outcomes with gilteritinib-containing triplets, preliminary data from the phase 2 NCT03661307 suggest the promising efficacy of quizartinib + DEC + VEN combination, with all five N/D patients achieving CR, with 4 of 5 achieving MRD-negativity by FLT3 PCR and 2 of 5 by flow cytometry [68]. The quizartinib-containing triplet has also been shown to have high activity in R/R patients with prior exposure to FLT3-inhibitors (78% previously treated with gilteritinib) [69]. Future direct, comparative studies will be required to determine whether gilteritinib or quizartinib is superior in specific genetic subpopulations.

As previously stated, TP53 and RAS pathway mutations are a significant major resistance factor to VEN-based regimens. Preliminary findings of the phase 1/2 APTIVATE (NCT03850574) trial that enrolled R/R FLT3-wild-type, TP53- and RAS-mutant AML patients show that tuspetinib, a new multikinase inhibitor, is well tolerated and increases survival of this cohort [70, 71]. We are also waiting for results from tuspetinib + AZA + VEN arm.

## IDH1- and IHD2-inhibitors

**Fig. 2:**
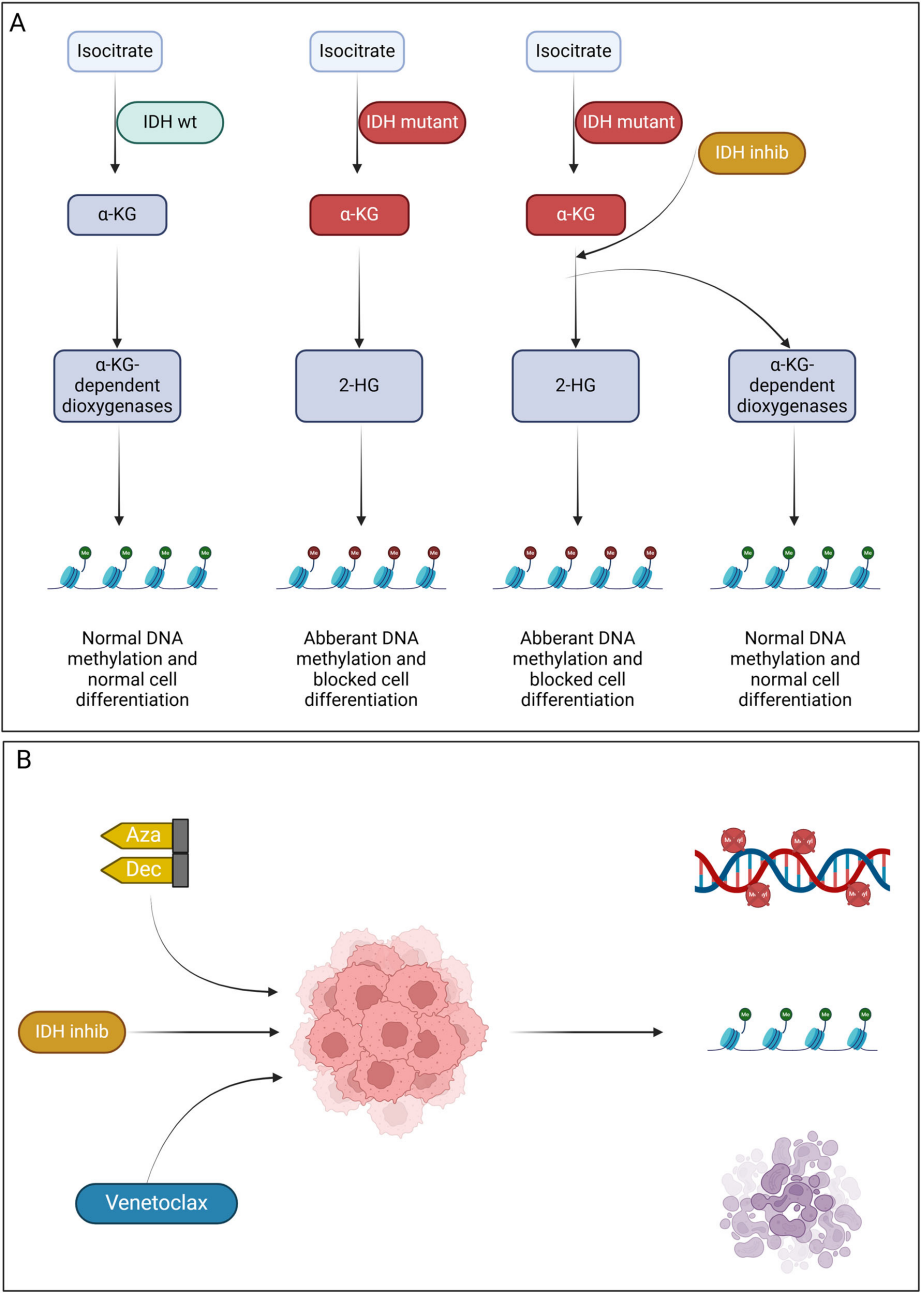
Epigenetic regulators that can serve as therapeutic strategies. **A** IDH role in DNA methylation. While wild-type (WT) IDH leads to normal DNA methylation and physiological production of alfa-ketoglutarate, mutant forms of IDH can lead to aberrant secretion of 2-hydroxy-glutarate (2-HG) and modified DNA methylation leading to a block in cell differentiation. The use of IDH-inhibitors in IDH mutant cells restores the normal DNA methylation pattern **B** The possible use of a triplet therapy including venetoclax, hypomethylating agents and IDH-inhibitors. The outcome would be a hypomethylated DNA, normal methylation due to IDH-inhibition and triggered apoptosis.

Either HMA + VEN or AZA + an IDH-inhibitor (Fig. 2) are currently the preferred options for elderly N/D patients unfit for IC. Whether AZA + VEN vs AZA + an IDH-inhibitor *vs* the HMA + IDH-inhibitor + VEN triplet is best for these patients remains an important clinical question. Furthermore, there is no evidence to suggest that frontline AZA + VEN followed by IDH-inhibitor + AZA in case of relapses is superior to the reverse order for IDH1/2 mutated AML. The DATA-I trial (NCT05401097) is currently recruiting subjects to answer this question [72].

The phase 1/2 NCT03471260 trial was the first study to demonstrate increased efficacy of ivosidenib + VEN + -AZA combination. The triplet led to higher median PFS (not reached, 95% CI 3.9-not reached vs 11 months, 95% CI 22.9–not reached, *P* = 0.058), but without significant difference in MRD-negative CR rates (75 vs 50%), IDH1 clearance (86 vs 43%) and OS (42.1 months vs not reached, *P* = 0.13). FLT3-TKD, N/KRAS, NF1, PTPN11, JAK/STAT, KIT, and CSF3R mutations have been associated with worse outcomes. MRD-negativity rate increased with increasing number of cycles, median IDH1-clearance occurred in cycle 4 and MRD-positivity in previous cycles has not been associated with worse outcomes, suggesting that MRD testing in later cycles might confer higher prognostic value [73]. The latest trial update indicates that the triplet may result in better long-term outcomes, although more follow-up and comparative, prospective trials are required [74].

Enasidenib, an IDH2-inhibitor, proves as well superior when combined with VEN. NCT04092179 investigated safety and efficacy in R/R patients and achieved an ORR of 70% with a median OS of 9.4 months (95% CI, 8.2 - not reached). R172 mutated patients achieved better response rates compared to R140 (ORR of 83 vs 55%) [75].

All-oral combinations may include IDH-inhibitors as well. The NCT04774393 combines ASTX727 + VEN with ivosidenib or enasidenib for IDH-mutated AML patients unfit for IC. Preliminary data showed high efficacy with 91% of N/D and 67% of R/R patients achieving MRD-negativity by flow cytometry [76]. A pooled analysis that compared ivosidenib + VEN + AZA vs the all-oral triplets showed no significant difference in terms of outcomes and safety [77].

The phase 1/2 NCT06445959 and NCT04603001 studies are investigating novel-generation IDH-inhibitors for R/R IDH-mutated AML not eligible for IC, such as olutasidenib or the dual IDH1- and IDH2-inhibitor crelosidenib. We are awaiting the first preliminary results.

## Menin-inhibitors

Menin inhibitors are a novel class of targeted therapies designed to disrupt the interaction between menin and NPM1, MLL1, NUP98, or KMT2A mutated proteins. The landscape of AML treatment is changing, with menin-inhibitors providing a less toxic option to classic therapies. These drugs may lead to responses in heavily pretreated populations, too. Currently, only preliminary data are available and only in R/R AML. The KOMET-007 trial (NCT05735184) investigated ziftomenib + AZA + VEN and achieved CR in 9 of 11 NPM1-mutated and 5 of 13 KMTA2-rearranged AML patients. Prior VEN exposure did not seem to influence efficacy [78]. Additionally, the ZiVA trial (NCT06397027) is evaluating ziftomenib + AZA + VEN in children and young adults (ages 2–30) with lower VEN exposure (14 days/cycle) [79]. SAVE (NCT05360160) investigated the all-oral revumenib + ASTX727 + VEN triplet achieving an ORR of 88% (23/26) with 17/23 patients reaching MRD-negativity by flow cytometry [80]. Revumenib + AZA + VEN is also tested in young (<30 years) R/R patients in the NCT06177067 trial. New generation menin-inhibitors, such as bleximenib or emilumenib, showed high synergism with VEN in vivo [81]. Clinical studies (NCT05453903, NCT04752163) for patients with R/R AML have been started to investigate these novel agents with an AZA + VEN backbone.

Currently, the KOMET-007 trial is the only one comparing the “7 + 3” IC regimen with ziftomenib to the ziftomenib + AZA + VEN triplet in N/D patients. Preclinical evidence suggests that menin inhibitors combined with FLT3-inhibitors + VEN might be one of the most effective and active therapies for N/D AML patients [82]. Prospective trials to investigate this triplet are warranted.

MRD-based addition of menin-inhibitors may also be a feasible approach in the future. The phase 2 NCT06284486 trial evaluates consolidation with the revumenib + VEN doublet in case of MRD-positivity (based on flow cytometry) following IC or low-intensity induction therapies [83].

## P53 reactivators and MDM2-inhibitors

MDM2 is an E3 ubiquitin ligase that binds to p53, causing its ubiquitination and proteasomal destruction. In AML, MDM2 overexpression or hyperactivity results in a functional p53 deficit, allowing leukemic cells to survive and proliferate [84]. MDM2 inhibitors (idasanutlin, navtemadlin and siremadlin) are molecules designed to disrupt the interaction between p53 and MDM2 and restore its tumor suppressive effects. Combination of p53 reactivators with VEN is a promising approach to increase the survival of patients with TP53 wild-type, R/R AML.

The phase 2 NCT02670044 investigated the combination of idasanutlin with VEN in R/R subjects ineligible for IC. The CR rate of the doublet, according to preliminary data, was 26% (13/50) with a median OS of 5.1 months (95% CI, 3.4–7.3). Patients with IDH1/2 or RUNX1 mutations achieved higher CR rates even in the presence of TP53 mutations. N/KRAS, FLT3, CBL, NF1, and PTPN1 mutations were associated with worse outcomes. Another limitation of the idasanutlin + VEN doublet may be the selective pressure for preexisting TP53-mutated clones [85]. Siremadlin, a novel-generation MDM2 inhibitor, has also been tested for R/R AML in combination with VEN in the NCT03940352 trial. Preliminary data reported four patients of ten achieving CR without any significant toxicities, cytopenias and febrile neutropenia being the most common adverse events [86]. The NCT05155709 trial is also testing siremadlin in a triplet combination with AZA and VEN for N/D patients unfit for IC without any TP53 mutations [87].

Eprenetapopt (APR-246) is a first-in-class small-molecule p53 reactivator that induces apoptosis also in TP53-mutated leukemic cells and could be the first FDA-approved targeted therapy for TP53-mutated AML. The NCT04214860 trial reported impressive outcomes with an ORR of 64% (25/39) and a median OS of 7.3 months (95% CI 5.6–9.8) without any treatment-related mortality. An indirect comparison of N/D patients revealed that the triplet achieved higher rates and longer-lasting responses than AZA + VEN alone [88].

## Cyclin-dependent kinase (CDK)-inhibitors

Dysregulated CDK activity is involved in leukemogenesis, particularly through its role in driving proliferation, evading apoptosis, and maintaining aberrant transcriptional programs. CDKs have emerged as attractive targets in AML to disrupt these pathological processes, restore cell cycle control and induce apoptosis. Upregulation of MCL-1, an antiapoptotic protein, plays a crucial role in the development of VEN resistance in AML. Alvocidib, a CDK9-inhibitor, downregulates MCL-1, and previous studies indicated that it synergizes with VEN [89]. The phase 1 NCT03441555 study aimed to evaluate the safety and preliminary efficacy of the alvocidib-VEN combination in adults with R/R AML. The study noted that responses (only 20% of patients) were observed primarily in patients with no prior exposure to VEN and overall, concluded that the combination did not yield sufficient efficacy to warrant further investigation [90]. Voruciclib is a new CDK9-inhibitor that led to responses in 10 of 32 (31%) patients in the NCT03547115 trial. However, only two of them obtained CR [91]. Triplet combinations on an AZA + VEN backbone are being investigated for many novel generation, potent CDK9-inhibitors that may improve outcomes. Preliminary data from the Chinese phase 2 trial NCT06532058 showed that 6 of the 18 evaluable patients achieved CR, with an ORR of 72.2% and 3 obtained MRD-negativity. The combination appears to induce responses in TP53-mutated AML as well [92]. Further evaluation is warranted.

## Selective inhibitors of nuclear export (SINEs)

Selinexor is a first-in-class selective inhibitor of nuclear export that targets exportin-1 (XPO1), a nuclear transport protein required for cellular homeostasis. By inhibiting XPO1, selinexor promotes the nuclear retention and activation of tumor suppressor proteins such as p53 while decreasing levels of antiapoptotic proteins, including MCL-1 [93]. This dual mechanism of action makes selinexor a promising agent to combine with VEN. Preliminary data of the phase 1 NCT05736965 trial reported high efficacy of the selinexor + AZA + VEN triplet in N/D patients ineligible for IC, with an ORR of 90% (18/20) and 16/20 achieving CR, with 3 patients being MRD-negative [94]. Several trials are currently enrolling R/R patients as well to assess the efficacy of selinexor combined with AZA + VEN. Eltanexor, a second-generation compound, is also tested with AZA + VEN for R/R AML in the phase 1 NCT06399640. We are waiting for the first preliminary results.

## Other targeted therapies

Recent advancements in the understanding of leukemogenesis have driven the development of novel targeted therapies, including tyrosine kinase inhibitors and agents targeting critical signaling pathways and epigenetic regulators. Table 3 summarizes the studies with targeted therapies that we identified. RAS pathway mutations are frequently associated with VEN resistance.

According to preclinical data, mitogen-activated protein kinase (MAPK)-inhibitors, such as trametinib or cobimetinib synergize with VEN and overcome VEN resistance [95]. NCT04487106 is a phase 1 trial that investigated the efficacy of trametinib in combination with VEN in R/R AML patients. Unfortunately, there was no improvement in outcomes, and the trial was terminated [96]. Similar results have been observed in NCT02670044 with cobimetinib [97]. Bromodomain and extraterminal (BET) family proteins are epigenetic regulators that play a role in leukemogenesis by upregulating antiapoptotic molecules. BET-inhibitors, such as mivebresib, have been tested in combination with VEN to more efficiently decrease the levels of BCL-xL, BCL-2 and upregulate proapoptotic/sensitizer peptides BIM and PUMA, which eventually would lead to higher apoptotic rates of leukemic cells. However, the NCT02391480 trial failed to demonstrate the efficacy of the combination [98].

Pevonedistat, a first-in-class inhibitor of the NEDD8-activating enzyme, disrupts the neddylation process, leading to decreased survival of leukemic cells through mechanisms such as reduced NF-κB activity and increased apoptosis. This approach aims to enhance the therapeutic potential of AZA + VEN. Results of the NCT03862157 trial showed a CR rate of 66% in the secondary AML cohort, with a median OS of 8.1 months. Therefore, the triplet combination demonstrates promising efficacy in secondary AML and warrants further evaluation [99]. In R/R AML, high efficacy, although in a small number of subjects, was demonstrated in five of seven patients, achieving CR with four subjects reaching MRD-negativity by flow cytometry. These findings suggest that adding pevonedistat to AZA + VEN might improve outcomes in heavily pretreated patients, and prospective trials assessing efficacy in specific genetic subgroups are needed [100].

Inflammation, oncogenesis, and cancer cell survival are all driven, among others, by interleukin-1 receptor-associated kinase 4 (IRAK4). An isoform of IRAK4 is overexpressed due to mutations in the splicing factors SF3B1 and U2AF1, which have been associated with worse outcomes in AML. The NCT04278768 trial investigated the efficacy of a novel IRAK4-inhibitor, CA-4948, in combination with AZA + VEN for R/R AML patients. Preliminary data show that two of five patients with spliceosome mutations achieved long-standing CR and were able to proceed to transplant [101].

Uproleselan (GMI-1271) is a new, first-in-class inhibitor of E-selectin, a cell adhesion protein that is essential in the tumor microenvironment of AML. E-selectin is overexpressed in the bone marrow vascular niche, where it promotes leukemic cell adhesion, chemoresistance, and immune evasion by promoting interaction between leukemic and bone marrow stromal cells. These interactions increase MRD rates, which contribute to relapse. The phase 1 NCT04964505 trial enrolled N/D AML patients unfit for IC and assessed the efficacy of uproleselan + VEN. CR has been achieved in 11 of 16 patients, nine of these also reached MRD-negativity. Therefore, the combination showed high antileukemic activity and needs further evaluation [102].

Ruxolitinib targets JAK2, a key kinase involved in the JAK-STAT signaling pathway, which is often dysregulated in hematologic malignancies, including AML. The combination of ruxolitinib and VEN is being explored in the NCT03874052 clinical trial. Preliminary data indicate that the combination is safe; nonetheless, only modest efficacy (10% CR rate and a median OS of only 3.7 months, 95% CI 2.3–6.5) has been described, with CD56-negativity being a predictive biomarker of response [103]. STAT-inhibitors, such as danvasertib or OPB-111077, are also investigated in the phase 1 NCT05986240 and NCT03063944 trials. Only one of seven patients achieved CR, and three died due to progressive disease [104].

## Immune checkpoint-inhibitors

Pembrolizumab, a programmed death-1 (PD1) inhibitor, combined with AZA or DEC + VEN, has been tested in both N/D and R/R patients in the NCT04284787 and NCT03969446. Preliminary data for N/D participants showed no increase in efficacy; nevertheless, worse outcomes were observed with the triplet, leading to early discontinuation of the trial [105]. Inhibition of PD1-ligand by avelumab in NCT03390296 did not bring any survival benefit either [106].

Magrolimab is a monoclonal antibody targeting CD47, designed to enhance macrophage-mediated phagocytosis of cancer cells by blocking the “don’t eat me” signal. NCT04435691 investigated the efficacy of magrolimab combined with the AZA + VEN backbone. High CR rates have been achieved in TP53-mutated N/D patients (*N* = 17) but with modest long-term outcomes (median OS = 7.6 months). CD47 upregulation and the inflammatory tumor microenvironment seem to be the main resistance mechanisms [107]. A phase 3 trial (ENHANCE-3, NCT05079230) to evaluate the combination has been designed but it was terminated due to increased risk of death in the magrolimab-arm.

Sabalotimab is an anti-TIM-3 monoclonal antibody, a new generation checkpoint-inhibitor, designed to enhance immune responses by targeting T-cell exhaustion. The STIMULUS-01 trial (NCT04150029) is currently enrolling N/D AML patients not suitable for IC to investigate sabalotimab in combination with AZA + VEN. According to initial preliminary data, the triplet is safe [108]. Additionally, we are awaiting the first efficacy reports.

Novel strategies to activate the immune system include downregulation of galectin-9 by the fully human monoclonal antibody, LYT-200. Preliminary data of the NCT05829226 trial showed that combination of LYT-200 with AZA + VEN in R/R AML led to 1 CR and 6 stable diseases of the 8 evaluable patients [109]. Further efficacy analysis is warranted.

In AML, a highly immunosuppressive tumor microenvironment, characterized by dysfunctional T-cells, regulatory T-cell expansion, and myeloid-derived suppressor cell infiltration, provides a rationale for targeting immune checkpoints. Preclinical data suggested that their combination with VEN may enhance antileukemic activity [110]. Currently available trials, however, fail to demonstrate any clinical benefit of adding VEN to checkpoint inhibitors. Another drawback worth considering is the substantial toxicity associated with checkpoint inhibitors, which mostly target barrier organs such as the gastrointestinal tract (colitis), skin (rash, dermatitis), lungs (pneumonitis), and liver (immune hepatitis). Up to 30% of patients may also present with endocrine system involvement, including thyroiditis, hypophysitis, and type 1 diabetes [111]. Combining checkpoint-inhibitors with other chemotherapy agents increased the risk of hypotension, myocarditis and arrhythmias [112]. The most concerning aspect, however, when combined with VEN, is the increased risk of developing hematologic toxicities, the most prevalent of which following checkpoint-inhibitor therapies are severe anemia (0.1–17%) and thrombocytopenia (1.2–2.5%) [113].

## Monoclonal antibodies and antibody-drug conjugates

Efficacy of the gemtuzumab ozogamicin, an anti-CD33 monoclonal antibody, and VEN doublet is currently tested in a phase 1 study NCT04070768. Efficacy of gemtuzumab + AZA + VEN proved a promising combination in NCT03390296, with 11 of 21 (52%) of treated R/R AML patients achieving CR or morphologic leukemia-free states with a median OS of 7.6 months. Poor-risk cytogenetics did not predict statistically different survival. Prior exposure to VEN, however, led to statistically significant decrease in responses (*P* = 0.02) [106].

Cusatuzumab is an anti-CD70 monoclonal antibody, another potential immunotherapy candidate, that targets leukemia stem cells and enhances anti-tumor immunity in AML. The phase 1 CULMINATE trial (NCT04023526) demonstrated modest efficacy of cusatuzumab in combination with AZA alone [114]. Thus, two other trials (NCT04150887 - ELEVATE and NCT06384261 - OV-AML-1231) have been initiated to test the antibody with AZA + VEN in N/D AML patients ineligible for IC. Preliminary data demonstrate an improved response rate (CR in 77.3%, *N* = 44) and high MRD-negativity rate (47% of the responders) [115].

Antibody-drug conjugates in AML, such as lintuzumab-225Ac, pivekimab, and tagraxofusp, represent novel immune therapies designed to deliver cytotoxic agents directly to leukemia cells, improving specificity and reducing off-target effects. Preclinical models demonstrated VEN resistance may be overcome by downregulation of MCL-1 by lintuzumab-225Ac, a monoclonal antibody targeting CD33 coupled with actinium-225 (225Ac), a potent cytotoxic, alpha-emitting radionuclide [116]. The phase 1/2 NCT03867682 trial investigated this combination and preliminary data showed high activity, two of three patients achieving morphologic leukemia-free status [117].

The first-in-class tagraxofusp and pivekimab sunirine are antibody-drug conjugates that target CD123. While tagraxofusp is linked to a diphtheria toxin, pivekimab sunirine contains a DNA alkylator payload (indolinobenzodiazepine pseudodimer). Although further research and head-to-head comparisons are necessary, preliminary results suggest that tagraxofusp combined with AZA + VEN improves outcomes in high-risk AML patients, particularly those with TP53 mutations. The phase 1 NCT03113643 trial reported achievement of CR in 69% of the overall population and 54% of TP53-mutated AML subjects (*N* = 26). Median OS for TP53 wild-type patients was not yet reached, with a median PFS of 13.3 months (95% CI, 8.6-not reached), while in the case of TP53-mutated AML, median PFS was 5.1 months (95% CI, 1.8-not reached) with median OS of 9.5 months (95% CI, 1.8-not reached), respectively. MRD-negativity by flow cytometry has been reached in four of seven TP53-mutated patients (57%) in CR. There have been no significant safety issues reported, with the exception of reversibly elevated liver enzymes. CD123 expression levels were not correlated with the duration or depth of response [118]. Subjects are now being recruited for a phase 2 trial (NCT06456463) with a modified treatment schedule (tagraxofusp is administered on days 4–6 instead of 1–3, and VEN is administered for 28 days instead of 21 days). Pivekimab sunirine on an AZA + VEN backbone achieved similar response rates for N/D patients unfit for IC. Of the 26 of 50 (52%) of patients in CR, 19 (73%) achieved MRD-negativity. No PFS or OS data have been published yet [119].

Other targetable antigens in AML include CD47, death receptor 5 and nectin-1. These surface molecules are involved in leukemic cell survival, immune evasion, and proliferation. Table 4 summarizes clinical trials investigating monoclonal antibodies against the aforementioned antigens. No data on safety or efficacy has been published yet.

## Immune cell-engagers and adoptive cellular therapies

Adoptive cellular therapies, including chimeric antigen receptor (CAR) T-cells and T-cell receptor (TCR)-engineered cells, have shown limited efficacy in AML due to challenges such as heterogeneous antigen expression, an immunosuppressive microenvironment, and relapse mechanisms. NK-cell-based adoptive cellular therapies, however, demonstrated favorable safety profiles and encouraging efficacy in AML, with response rates ranging from 25 to 88% even in patients unfit for allotransplantation [120]. Therefore, allogeneic NK-cells administered with AZA + VEN may represent an alternative approach to improve survival in elderly patients. ADVENT-AML (NCT05834244) is the first trial to investigate cellular therapy in a frontline setting in AML. Additionally, this research would provide information on the efficacy of lymphodepletion with AZA + VEN or the synergy shown between adoptive therapies and VEN. NCT06152809 is investigating the impact of allogeneic NK-cells in combination with AZA + VEN post-induction, during consolidation. No results have been published yet.

SAR443579, a natural killer (NK)-cell engager with three binding domains, one to CD123 on the surface of leukemic cells and two others to NKp46 and CD16a on NK-cells, is another novel strategy to overcome the immunosuppressive niche by activating immune cells via direct blast-immune cell crosslinking. NCT06508489 is currently recruiting patients to test this agent in combination with AZA + VEN for N/D AML.

## The role of biomarkers to predict responses to venetoclax-based combinations

The European LeukemiaNet (ELN) 2022 risk classification proved to be efficient in stratifying patients given IC [7]. Döhner et al., however, reported that this classification system is not accurately prognostic of patients receiving HMA + VEN [121]. To improve the prognostic scores of patients receiving HMA + VEN, the Beat-AML score added a mutation score based on the presence of IDH2, KRAS, MLL2, and TP53 mutations, redefining AML patients who were earlier categorized as adverse risk based on ELN 2022 in an intermediate (−1 or 0 point) and an adverse risk group (1+ points). KRAS, MLL2, and TP53 mutations were unfavorable (+1 point each); however, IDH2 was regarded as an independent positive prognostic factor (−1 point) [122]. Another ELN 2024, four-gene (FLT3-ITD, KRAS, NRAS, and TP53) molecular prognostic risk signature (mPRS) has also been developed and classifies patients into three prognostic groups [123]. A retrospective study at MD Anderson Cancer Center in Houston has confirmed that the median PFS and OS are considerably different using the ELN 2024 mPRS: 19 and 30 months for the higher benefit, 8 and 12 months for the intermediate benefit (FLT3-ITD, NRAS or KRAS-mutated), and 4 and 5 months for the lower benefit (TP53-mutated) groups (*P* < 0.001) [124]. While the Beat-AML and the ELN 2024 mPRS are predicting survival, the Mayo score has been developed as a predictor of treatment response and categorized patients in four molecular signatures based on a six-gene panel (NPM1, IDH2, DDX41, TP53, RUNX1, and FLT3-ITD). In the Mayo risk model, survival was superior in patients with IDH2 R172K vs. R140Q mutations (not reached vs. 19.2 months, *P* = 0.02) [125]. KMT2A rearrangements, presence of a PTPN11 mutation, monocytic or secondary AML and male gender have also been identified as risk factors for a worse prognosis [126]. Novel prognostic scores that incorporate MRD-status, although not currently used in the clinic, are also needed in the future.

## Conclusion

Venetoclax has emerged as a game changer in the treatment landscape of AML. VEN combined with targeted therapies or epigenetic regulators like HMA, FLT3-, IDH-, or menin-inhibitors, as well as immunotherapies (monoclonal antibodies, antibody-drug conjugates, or adoptive cellular therapies) not only improves efficacy but holds the potential to overcome resistance mechanisms that frequently limit monotherapy. Therefore, basic and translational research to identify novel resistance mechanisms is vital.

VEN-based combination therapies are tailored to balance efficacy with safety. Which combination is best and whether the addition of multiple agents is beneficial, and if so, the optimal dose, treatment duration and disease subtypes remain to be determined. Development of a mutation-guided therapeutic pathway is one of the most significant challenges in modern clinical practice due to the many important questions that remain to be answered. For instance, for patients with FLT3-mutations alone, the experience with triple therapy emerging from clinical trials as well as from real-world experience is that the use of gilteritinib + HMA + VEN is very toxic and the high degree of cytopenias is that makes the combination difficult to use. Perhaps using the lower duration of VEN (7 or 14 days) may be sufficient to make it more tolerable. An alternative approach is to use the drugs sequentially. For instance, given the fact that FLT3-mutant clones at diagnosis represent a relatively small fraction of the malignant cells, we might initiate HMA + VEN for 14 or 21 days and start gilteritinib only after day 21 or 28. Subsequent cycles can use either 7 or 14 days of VEN. If a patient has both FLT3-ITD and IDH mutations, what shall one use for induction/consolidation is another unanswered problem. Generally, IDH-mutations confer resistance to FLT3-inhibitors and thus, a combo of FLT3-ITD + IDH mutations may be less likely to respond to FLT3-inhibitors, particularly at diagnosis. Thus, one could use FLT3-inhibitors only in consolidation/maintenance/relapse. On the other hand, IDH-mutant AML cells are more sensitive to VEN (IDH1 mutants are less so than IDH2). Thus, for IDH1-mutant AML, one may consider using IDH1-inhibitors rather than HMA + VEN, while for the IDH2-mutant disease, perhaps the use of HMA + VEN may be more likely to offer clinical benefits. The bottom line is that there is no standardized approach for these clinical situations. Perhaps novel clinical trial designs and artificial intelligence algorithms should predict the best sequence of therapies based on the type of mutation, the variant allele frequency of mutations, the molecular interactions between the signaling programs activated downstream of the mutations, but also based on drug and reimbursement availability in various health care systems, as well as clinical toxicities.

The increased turnaround time for genomic and other molecular tests creates a further clinical dilemma about whether empiric/early treatment should be undertaken. A comprehensive registry-based analysis of 854 AML patients treated with VEN-based combinations, however, indicates that there is no significant difference in response rates or mortality when treatment is initiated within the first 10 days of diagnosis or later. Therefore, delaying treatment for optimal and personalized treatment selection may become a viable approach in the clinic since better clinical monitoring and risk analysis may be performed [127].

While the clinical outcomes are encouraging, challenges remain, particularly regarding the identification of predictive biomarkers to guide patient selection. The use of novel prognostic scores, such as the Beat-AML, the Mayo or the ELN 2024 classifications, are an important step towards precision therapy. Future research should prioritize well-designed clinical trials, real-world studies, and mechanistic insights to refine these combinations and expand their applicability. Further trials are needed to assess optimal duration of treatment, but we are optimistic that future clinical trials will confirm that decreasing VEN exposure to 14 or 21 days, or even to 7 days, and HMA exposure to 5 or 3 days provides a significant clinical benefit [121].

Furthermore, there is now an unmet need for the development of novel-generation BCL-2 inhibitors that can target both MCL-1 and BCL-xL without causing any major cardiac or hematologic toxicity. WH25244, for instance is a novel BCL-2 and BCL-xL inhibitor that spares platelets and acts on mutant, hyperphosphorylated BCL-2 molecules that showed high cytotoxicity in vitro. Further preclinical and clinical studies are needed to confirm its efficacy [128].

Overall, the combinations presented in this manuscript represent a paradigm shift in AML management, paving the path for longer-lasting remissions and better patient outcomes. Continued innovations and collaboration will be essential to harness the potential of declaring this currently incurable disease a curable one.
